# Optimal collimator rotation based on the outline of multiple brain targets in VMAT

**DOI:** 10.1186/s13014-018-1039-5

**Published:** 2018-05-09

**Authors:** Jung-in Kim, Beom Seok Ahn, Chang Heon Choi, Jong Min Park, So-Yeon Park

**Affiliations:** 1 Department of Radiation Oncology, Seoul National University Hospital, Seoul, Republic of Korea; 2 Institute of Radiation Medicine, Seoul National University Medical Research Center, Seoul, Republic of Korea; 3 Biomedical Research Institute, Seoul National University College of Medicine, Seoul, Republic of Korea; 4 Department of Radiation Oncology, Seoul National University Boramae Medical Center, Seoul, Republic of Korea; 5 Center for Convergence Research on Robotics, Advance Institutes of Convergence Technology, Suwon, Republic of Korea; 6 Department of Radiation Oncology, Veterans Health Service Medical Center, Seoul, Republic of Korea

## Abstract

**Background:**

The aim of this study was to investigate the dosimetric quality in volumetric modulated arc therapy (VMAT) plans with optimal collimator angles that can represent the outline of multiple brain targets.

**Methods:**

Twenty patients with multiple target volumes in the brain cases were selected retrospectively. To better represent the outline of the multiple brain targets, four conformal arc plans were generated for each patient using one full arc with four collimator settings. The optimal collimator angles calculated from the integrated multi-leaf collimator (MLC) aperture that had the smallest aperture size for certain collimator settings of the conformal arc plan were selected. VMAT plans with the optimal collimator angles with angular sections of 40° and 60° (Colli-VMAT (40°), Colli-VMAT (60°)) were generated, followed by evaluation of field sizes, dose-volumetric parameters and total monitor units (MUs).

**Results:**

Patient-averaged values of field sizes for Colli-VMAT (40°) (111.5 cm^2^) were lowest and 1.3 times smaller than those for Std-VMAT (143.6 cm^2^). Colli-VMAT plans improved sparing of most normal organs but for brain stem and left parotid gland. For the total MUs, the averaged values obtained with the Colli-VMAT (40°) (390 ± 148 MU) were smaller than those obtained with the Std-VMAT (472 ± 235 MU).

**Conclusions:**

The Colli-VMAT plans with smaller angular sections could be suitable in the clinic for multiple brain targets as well as for irregularly shaped targets. Determination of the optimal collimator rotation generally showed good normal tissue sparing and MU reduction for multiple brain targets.

## Background

Volumetric modulated arc therapy (VMAT) can achieve highly conformal dose distribution to target volumes while sparing normal tissues, using intensity-modulated photon beams by simultaneous modulations of multi-leaf collimator (MLC) positions, gantry rotation speed, and dose-rate [1, 2]. Planning studies have consistently demonstrated that VMAT plans show equivalent dosimetric plan quality and usually reduce monitor unit (MU) usage compared to intensity-modulated radiation therapy (IMRT) [3–5]. Because of these reasons, VMAT has been widely used for various treatment sites clinically [6–11].

Collimator angle is an important parameter that affects dosimetric plan quality; however, the current technology does not allow collimator rotation during VMAT delivery, and thus, a single optimal collimator angle must be selected. The optimal collimator angle can be determined manually based on the user’s experience to consider target shape, size, and placement in the clinic. Several studies have reported that a collimator angle of 45° has been appropriate in most cases and acquired better dosimetric plan quality than other angles [1, 5, 12]. In Eclipse™ (Varian Medical Systems, Palo Alto, CA), the recommended collimator angles for VMAT plan optimization are collimator rotations of 10° (350°) and 30° (330°) for most cases [13].

In order to evaluate the effectiveness of dynamic collimator angle during VMAT delivery, Boer et al. adjusted the collimator and gantry angle for considering left-right prostate rotation [14]. They reported that the left-right prostate rotation could be compensated with this technique. Zhang et al. proposed a collimator trajectory optimization algorithm based on a principal component calculated from a beam’s-eye view (BEV) of the spinal cord [15]. These values of principal component could provide the long axis of the cord and then suitable MLC direction could be selected. These studies have shown dosimetric improvement compared to VMAT plans with a fixed collimator angle.

In a previous study, we analyzed the dosimetric effects of optimal collimator angles at each sectional arc in the VMAT for an irregularly shaped target in abdominal, head and neck, and chest cases [16]. In that study, the optimal collimator angles were calculated using an integrated MLC aperture from a conformal arc plan generated with a fixed collimator setting of 0°. Although we demonstrated considerable improvements of dosimetric plan quality and MU efficiency using the optimal collimator angles, the integrated MLC apertures with a fixed collimator setting of 0° had limitations that could not fully express the shape of the targets defined by the MLCs [16]. For multiple brain targets, the conformal arc plans with a fixed collimator angle of 0° used in our previous study were not appropriate. If we can represent all the outlines of the targets using the MLC apertures regardless of shape, size, and placement of the targets, the optimal collimator angles calculated from these integrated MLC apertures might have better potential to improve the dosimetric plan quality or MU efficiency.

In this study, we attempted to generate the integrated MLC apertures from various collimator settings, and then calculated the optimal collimator angles using these integrated MLC apertures for multiple brain targets. We tested the performance of this technique and then compared the dosimetric plan quality and total MUs to those obtained in our previous study, as well as the conventional VMAT plans with a fixed collimator angle (Std-VMAT).

## Methods

### Patient selection

Twenty patients with multiple target volumes in the brain were retrospectively selected. All had been previously treated with radiotherapy at our institution. An approval for this study was obtained from the institutional review board (IRB No. 1612–013-811). The maximum number of multiple target volumes was 5 and maximum distance between multiple target volumes was 11.9 cm with averaged value of 6.8 cm. The 20 patients were given 5 to 28 fractions with prescription doses ranging from 15.0 Gy to 50.4 Gy. The patient characteristics are summarized in Table 1.

**Table 1:** Patient characteristics

Patient ID	The number of target volumes	Maximum distance b/w target volumes (cm)	Prescription dose (Gy)	Fraction
1	2	6.5	30.0	10
2	2	5.6	30.0	10
3	2	5.4	30.0	10
4	5	8.7	18.0	6
5	4	11.9	20.0	5
6	3	9.0	18.0	10
7	2	7.0	21.6	12
8	3	7.6	45.0	25
9	2	10.4	35.0	10
10	4	11.3	15.0	5
11	2	6.0	36.0	20
12	2	8.2	36.0	12
13	2	4.0	45.0	25
14	2	7.5	50.4	28
15	2	4.2	45.0	25
16	2	5.0	50.4	28
17	2	4.0	27.0	9
18	2	5.4	15.0	5
19	2	4.1	50.4	28
20	2	4.1	50.4	28

### Determination of optimal collimator angles

In this study, the commercial treatment planning system, Eclipse™ version 10 (Varian Medical Systems, Palo Alto, CA), was used. In order to obtain the MLC apertures for the multiple brain targets and then generate the integrated MLC apertures within angular sections, four conformal arc plans were generated for each patient using one full arc with four collimator settings of 0°, 45°, 90°, and 135°, respectively. The number of control points (CPs) for each conformal arc plan was 500, which is the maximum value obtained with 0.72°/CP spacing. Four conformal arc plans were exported in DICOM-RT format from Eclipse™. The MLC positions represented as the outline of the target for each CP were obtained using an in-house program written in MATLAB (R2016a, Mathworks, Inc., Natick, MA).

In our previous study, we determined the optimal collimator angles with various angular sections using the conformal arc plans with a fixed collimator angle of 0° and then demonstrated that VMAT plans with the optimal collimator angles with angular sections of 40° and 60° (Colli-VMAT_Pre_ (40°), Colli-VMAT_Pre_ (60°)) reduced the total MUs and improved the sparing of normal organs, compared to Colli-VMAT_Pre_ (90°), Colli-VMAT_Pre_ (120°), and Std-VMAT [16]. Std-VMAT plan was one full-arc VMAT plans with a fixed collimator angle, having an angular section of 360°. The fixed collimator angle for Std-VMAT plans was calculated using the same procedure as that for calculating the optimal collimator angle of Colli-VMAT_Pre_ plans.

**Fig. 1:**
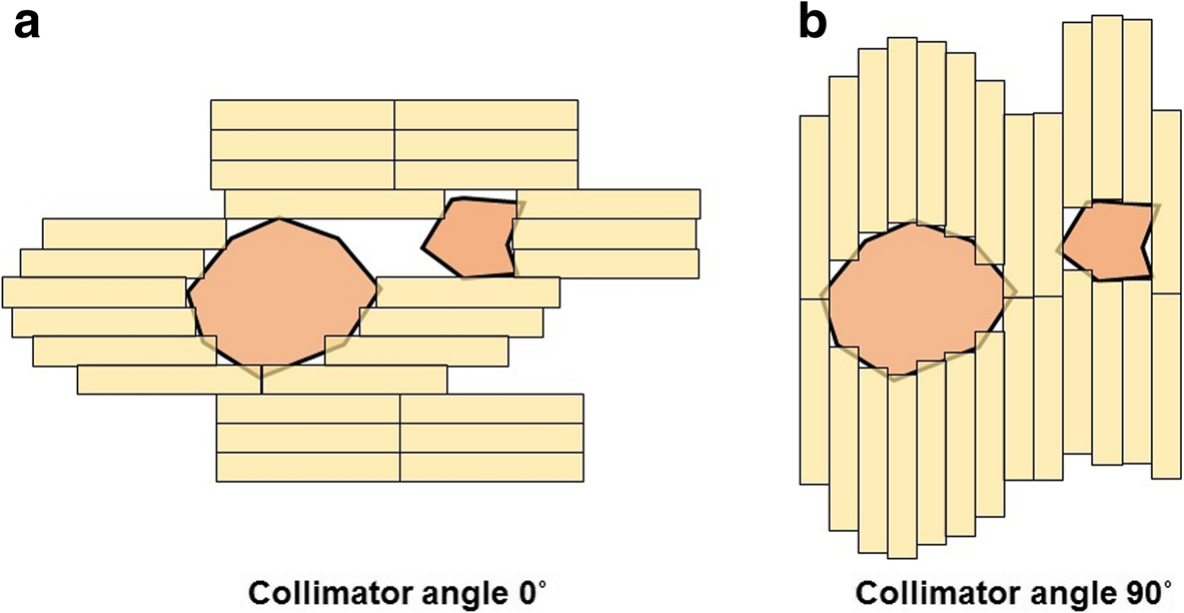
Beam’s-eye view (BEV) of target volumes with multiple brain targets fitted by multi-leaf collimators (MLCs) at collimator settings of 0° (**a**) and 90° (**b**)

In this study, angular sections of 40° and 60° were chosen while the VMAT plans had sectional arcs (partial arc field) of 9 and 6 according to the angular sections of 40° and 60°, respectively. The integrated MLC apertures were determined by the MLC positions having the largest gap within the angular section to cover the target volumes based on the beam’s-eye view (BEV). For each of the sectional arcs in the VMAT plan, the aperture sizes of the integrated MLC apertures from the conformal arc plans with four collimator settings of 0°, 45°, 90°, and 135° were compared. Of these collimator settings, the integrated MLC apertures that had the smallest aperture size for a certain collimator setting of the conformal arc plan were selected. Figure 1 demonstrates the integrated MLC apertures for different collimator settings of the conformal arc plans and proper collimator setting can represent the shape of multiple brain targets exactly within the angular section.

**Fig. 2:**
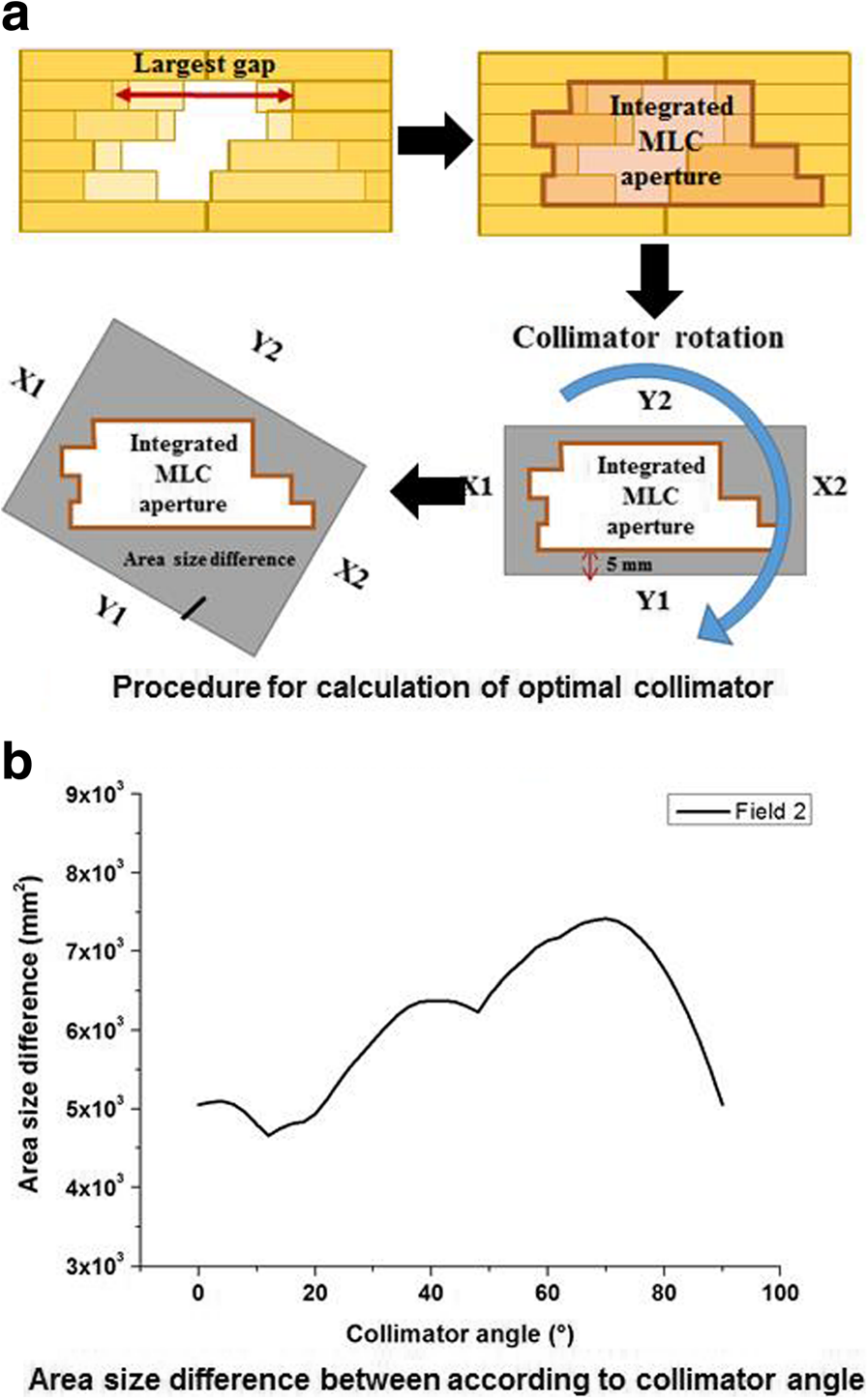
Detailed procedure for generation of integrated multi-leaf collimators (MLC) aperture and determination of optimal collimator angle (**a**) and an example of area size difference between the integrated MLC aperture and collimator settings (**b**) with 5-mm margins to the integrated MLC aperture according to collimator angles in Field 2 (Sectional arc 2)

With the integrated MLC apertures chosen in this study, we obtained the optimal collimator angles by minimizing the area size difference between the integrated MLC aperture and the collimator settings. As the collimator rotated, the collimator settings had 5-mm margins to the integrated MLC aperture. Figure 2 describes the more detailed procedure to calculate optimal collimator angle and an example of area size difference between the integrated MLC apertures and collimator settings. This process was repeated to calculate the optimal collimator angle for each sectional arc. The VMAT plans suggested in this study according to the angular sections of 40° and 60° (Colli-VMAT (40°) and Colli-VMAT (60°)) were generated in Eclipse™. For comparison purposes, Colli-VMAT_Pre_ (40°), Colli-VMAT_Pre_ (60°), and Std-VMAT plans were generated.

In the same manner as our previous study [16], some of the calculated optimal collimator angles for sectional arcs were re-determined by adding 90° rotation to those calculated optimal collimator angles before VMAT plan optimization. The reason for adding 90° rotation is that the collimator angle added to 90° rotation could potentially reduce the burden of MLC control for modulating photon beam intensity owing to the maximum leaf span of the MLCs (15 cm). If the maximum distance in the integrated MLC apertures exceeded the maximum leaf span in the MLC direction, the collimator angles added to 90° were re-chosen as the optimal collimator angles to improve the target conformity.

### VMAT plans for multiple brain targets

All VMAT plans were created with 6 MV photon beams from Trilogy™ with Millennium™ 120 MLC (Varian Medical Systems, Palo Alto, CA). Every VMAT plan was optimized with the progressive resolution optimizer 3 (PRO3, ver. 10, Varian Medical Systems, Palo Alto, CA). To acquire better dosimetric plan quality, all VMAT plans were re-optimized using current dose distribution as the reference. The dose distributions were calculated by using the anisotropic analytic algorithm (AAA, ver. 10, Varian Medical Systems, Palo Alto, CA) with a calculation grid of 1 mm to remove the calculation grid dependency. All plans were normalized so that 95% of the prescription dose covered 100% of the target volume.

### Dosimetric analysis and evaluation

The dose-volume histogram (DVH) data was used to evaluate the dosimetric quality with respect to target coverage and dose received by normal organs. For the target volumes, the evaluated dose-volumetric parameters were the mean dose, maximum dose, minimum dose, dose received by at least 99% volume of the target volume (D_99%_), D_95%_, D_5%_, D_1%_, conformity index (CI), homogeneity index (HI), and gradient measure (GM). The CI, HI, and GM are defined as follows [2, 17–19]:

1 $$ Conformity\kern0.5em index\kern0.5em (CI)=\frac{Volume\kern0.17em of\kern0.17em reference\kern0.17em isodose}{Volume\kern0.17em of\kern0.17em target\kern0.17em volume}, $$

2 $$ Homogeneity\kern0.5em index\kern0.5em (HI)\kern0.5em =\kern0.5em \frac{D_{2\%}-{D}_{98\%}}{D_{50\%}}, $$

and

3 $$ Gradient\kern0.5em measure\kern0.5em (GM)\kern0.5em =\kern0.5em {R}_{50\% of\ presecription\ dose}-{R}_{presecription\kern0.17em dose}, $$

where the volume of *reference isodose* is the volume irradiated by 95% of the prescription dose, and *R*_*x*_ is the sphere radius of which the volume is the same as the volume of isodose of *x*.

For the normal organs, the following were calculated: the absolute volume of a normal brain without target volumes irradiated by at least 5 Gy (V_5 Gy_), V_12 Gy_, and V_15 Gy_; the mean dose of a normal brain without target volumes; the maximum dose of the spinal cord, brain stem, optic chiasm, right and left optic nerves, and right and left lenses; and the mean dose of the right and left parotid glands.

Averaged total MUs were compared and MU reduction was calculated to evaluate the relative delivery efficiency of each VMAT plan. The MU reduction is defined as

4 $$ \mathrm{MU}\ \mathrm{reduction}\ \left(\%\right)=\frac{MU_{Colli- VMAT}-{MU}_{Std- VMAT}}{MU_{Std- VMAT}}, $$

where MU_*colli-VMAT*_ and MU_*Std-VMAT*_ are total MUs for Colli-VMAT and Std-VMAT, respectively.

For multiple comparison statistics, the patient-averaged values of field sizes, DVH data, and total MUs were analyzed with one-way analysis of variance (ANOVA) with the Bonferroni correction and then Bonferroni-adjusted *p*-values were calculated. Significance was defined as *p* <  0.05 for 10 pairwise comparisons. All analysis was performed by SPSS version 14.0 (SPSS Inc., Chicago, IL).

## Results

### Field sizes for optimal collimator angle

After obtaining the optimal collimator angles for Std-VMAT plan, Colli-VMAT_Pre_ plans, and Colli-VMAT plans, field sizes according to these sectional arcs were calculated and averaged for each patient, as shown in Table 2. The patient-averaged values of the field size were 143.6 cm^2^ ± 60.8 cm^2^, 113.5 cm^2^ ± 49.7 cm^2^, 114.8 cm^2^ ± 50.9 cm^2^, 111.5 cm^2^ ± 48.8 cm^2^, and 115.1 cm^2^ ± 49.4 cm^2^ for Std-VMAT, Colli-VMAT_Pre_ (40°), Colli-VMAT_Pre_ (60°), Colli-VMAT (40°), and Colli-VMAT (60°), respectively. The *p*-values for one-way analysis of variance (ANOVA) with Bonferroni multiple comparison of the patient-averaged values of the field sizes are shown in Table 2. The patient-averaged values of the field sizes for Colli-VMAT_Pre_ (40°) and Colli-VMAT (40°) demonstrated improved results with an average of 26.5 and 28.8% reductions with statistical significance (*p* = 0.001 and *p* = 0.001, respectively), compared with Std-VMAT. For Colli-VMAT (40°) and Colli-VMAT (60°) plans, the values of the field sizes had decreasing tendencies when the values of angular sections became smaller with statistical significance (*p* = 0.015). When comparing Colli-VMAT_Pre_ (40°) to Colli-VMAT (40°), there were slight decreasing tendencies in the values of the field sizes, which were statistically significant in our current study (*p* = 0.023).

**Table 2:** Patient-averaged values of field sizes according to the optimal collimator angles, and *p* values for one way analysis of variance (ANOVA) with Bonferroni multiple comparison test of the patient-averaged values of field sizes

	Std-VMAT	Colli-VMAT_Pre_ (40°)	Colli-VMAT_Pre_ (60°)	Colli-VMAT (40°)	Colli-VMAT (60°)
Average (cm^2^)	143.6 ± 60.8	113.5 ± 49.7	114.8 ± 50.9	111.5 ± 48.8	115.1 ± 49.4
Bonferroni adjusted *p*-values					
Colli-VMAT_Pre_ (40°)	0.001	–	–	–	–
Colli-VMAT_Pre_ (60°)	0.005	–	–	–	–
Colli-VMAT (40°)	0.001	0.023	0.037	–	
Colli-VMAT (60°)	0.002	–	–	0.015	–

### Dosimetric analysis and evaluation

The patient-averaged values of dose-volumetric parameters of Std-VMAT plan, Colli-VMAT_Pre_ plans, and Colli-VMAT plans for multiple brain targets and normal tissues are shown in Table 3.

**Table 3:** Averaged values of dose-volumetric parameters

Structure	DV parameter^a^	Std-VMAT	Colli-VMAT_Pre_ (40°)	Colli-VMAT_Pre_ (60°)	Colli-VMAT (40°)	Colli-VMAT (60°)
Target volume	D_99%_^b^ (Gy)	28.8 ± 11.9	28.6 ± 11.6	28.7 ± 11.7	28.6 ± 11.6	28.7 ± 11.7
	D_95%_ (Gy)	29.4 ± 12.4	29.2 ± 12.2	29.3 ± 12.4	29.3 ± 12.2	29.4 ± 12.2
	D_5%_ (Gy)	32.3 ± 15.4	32.4 ± 15.1	32.3 ± 15.4	32.4 ± 15.1	32.2 ± 15.0
	D_1%_ (Gy)	32.6 ± 15.7	32.7 ± 15.2	32.6 ± 15.5	32.7 ± 15.2	32.4 ± 15.1
	Minimum dose (Gy)	21.4 ± 10.0	22.0 ± 10.1	21.4 ± 10.0	22.1 ± 9.9	21.1 ± 10.1
	Maximum dose (Gy)	33.2 ± 14.3.	33.1 ± 14.4	33.2 ± 15.3.	33.0 ± 15.4	33.1 ± 15.3
	Mean dose (Gy)	31.1 ± 15.0	31.4 ± 14.4	31.4 ± 15.0	31.4 ± 15.3	31.3 ± 15.4
	Conformity index	0.8 ± 0.1	0.8 ± 0.1	0.8 ± 0.1	0.8 ± 0.1	0.8 ± 0.1
	Homogeneity index	0.09 ± 0.02	0.10 ± 0.06	0.09 ± 0.03	0.10 ± 0.04	0.10 ± 0.02
	Gradient measure (cm)	1.3 ± 0.2	1.4 ± 0.1	1.3 ± 0.1	1.4 ± 0.1	1.3 ± 0.1
Normal brain - PTV	V_5 Gy_^c^ (cc)	776.6 ± 316.3	778.0 ± 320.1	783.4 ± 320.4	764.6 ± 318.2	766.0 ± 313.3
	V_12 Gy_ (cc)	454.5 ± 333.4	443.3 ± 340.7	448.7 ± 342.9	439.5 ± 341.1	441.1 ± 335.8
	V_15 Gy_ (cc)	357.3 ± 333.4	351.5 ± 340.7	356.7 ± 342.9	347.5 ± 341.1	348.5 ± 335.8
	Mean dose (Gy)	13.4 ± 9.9	13.3 ± 10.0	13.4 ± 10.0	13.2 ± 10.1	13.1 ± 9.7
Spinal cord	Maximum dose (Gy)	3.6 ± 2.0	3.2 ± 2.8	3.1 ± 2.2	1.8 ± 2.5	3.1 ± 2.9
Brain stem	Maximum dose (Gy)	26.6 ± 15.3	26.6 ± 16.6	26.6 ± 16.7	26.4 ± 16.5	26.3 ± 15.9
Optic chiasm	Maximum dose (Gy)	26.4 ± 11.5	25.7 ± 11.5	25.5 ± 11.6	25.0 ± 12.0	25.3 ± 11.3
Right optic nerve	Maximum dose (Gy)	19.6 ± 16.8	18.5 ± 16.3	18.4 ± 16.9	17.7 ± 16.8	18.0 ± 17.0
Left optic nerve	Maximum dose (Gy)	19.6 ± 17.5	17.8 ± 16.6	18.4 ± 17.0	17.6 ± 16.8	18.2 ± 17.5
Right lens	Maximum dose (Gy)	3.5 ± 2.2	3.4 ± 3.0	3.4 ± 2.8	3.0 ± 3.1	3.0 ± 2.0
Left lens	Maximum dose (Gy)	3.1 ± 1.9	3.0 ± 2.0	3.2 ± 2.3	2.8 ± 2.0	3.0 ± 1.7
Right parotid	Mean dose (Gy)	0.9 ± 1.2	0.8 ± 1.3	0.8 ± 1.2	0.7 ± 1.1	0.8 ± 1.2
Left parotid	Mean dose (Gy)	1.0 ± 1.2	1.1 ± 1.2	1.1 ± 1.0	1.1 ± 1.2	1.1 ± 1.0

^a^DV parameter = dose-volumetric parameter

^b^D_n%_ = dose received at least n% volume of a structure

^c^V_n Gy_ = absolute volume irradiated by at least n Gy of a structure

For multiple brain targets, the patient-averaged values of D_99%_, D_95%_, D_5%_, D_1%_, minimum dose, maximum dose, mean dose, conformity index, homogeneity index, and gradient measure for target volume were similar regardless of the VMAT plans, with no statistical significance in all the VMAT plans compared. For more detailed results, the patient-averaged values of dose-volumetric parameters for normal organs were obtained and the *p*-values for one-way analysis of variance (ANOVA) with Bonferroni multiple comparison of the patient-averaged values of dose-volumetric parameters were also calculated by comparing Std-VMAT, Colli-VMAT_Pre_ (40°), and Colli-VMAT (40°), and are listed in Table 4.

**Table 4:** *p* values for one way analysis of variance (ANOVA) with Bonferroni multiple comparison test of the patient-averaged values of dose-volumetric parameters

		Bonferroni adjusted *p*-values		
Structure	DV parameter^a^	Std-VMAT vs. Colli-VMAT_Pre_ (40°)	Std-VMAT vs. Colli-VMAT (40°)	Colli-VMAT_Pre_ (40°) vs. Colli-VMAT (40°)
Normal brain - PTV	V_5 Gy_^b^ (cc)	< 0.001	< 0.001	< 0.001
	V_12 Gy_ (cc)	0.002	0.023	0.036
	V_15 Gy_ (cc)	< 0.001	< 0.001	0.024
	Mean dose (Gy)	–	–	–
Spinal cord	Maximum dose (Gy)	0.025	0.018	0.015
Brain stem	Maximum dose (Gy)	–	–	0.048
Optic chiasm	Maximum dose (Gy)	–	0.043	0.033
Right optic nerve	Maximum dose (Gy)	0.025	0.035	0.016
Left optic nerve	Maximum dose (Gy)	0.023	0.036	–
Right lens	Maximum dose (Gy)	–	0.042	0.048
Left lens	Maximum dose (Gy)	–	–	–
Right parotid	Mean dose (Gy)	0.025	–	–
Left parotid	Mean dose (Gy)	–	–	–

^a^DV parameter = dose-volumetric parameter

^b^V_n Gy_ = The absolute volume of a structure irradiated by at least n Gy

**Fig. 3:**
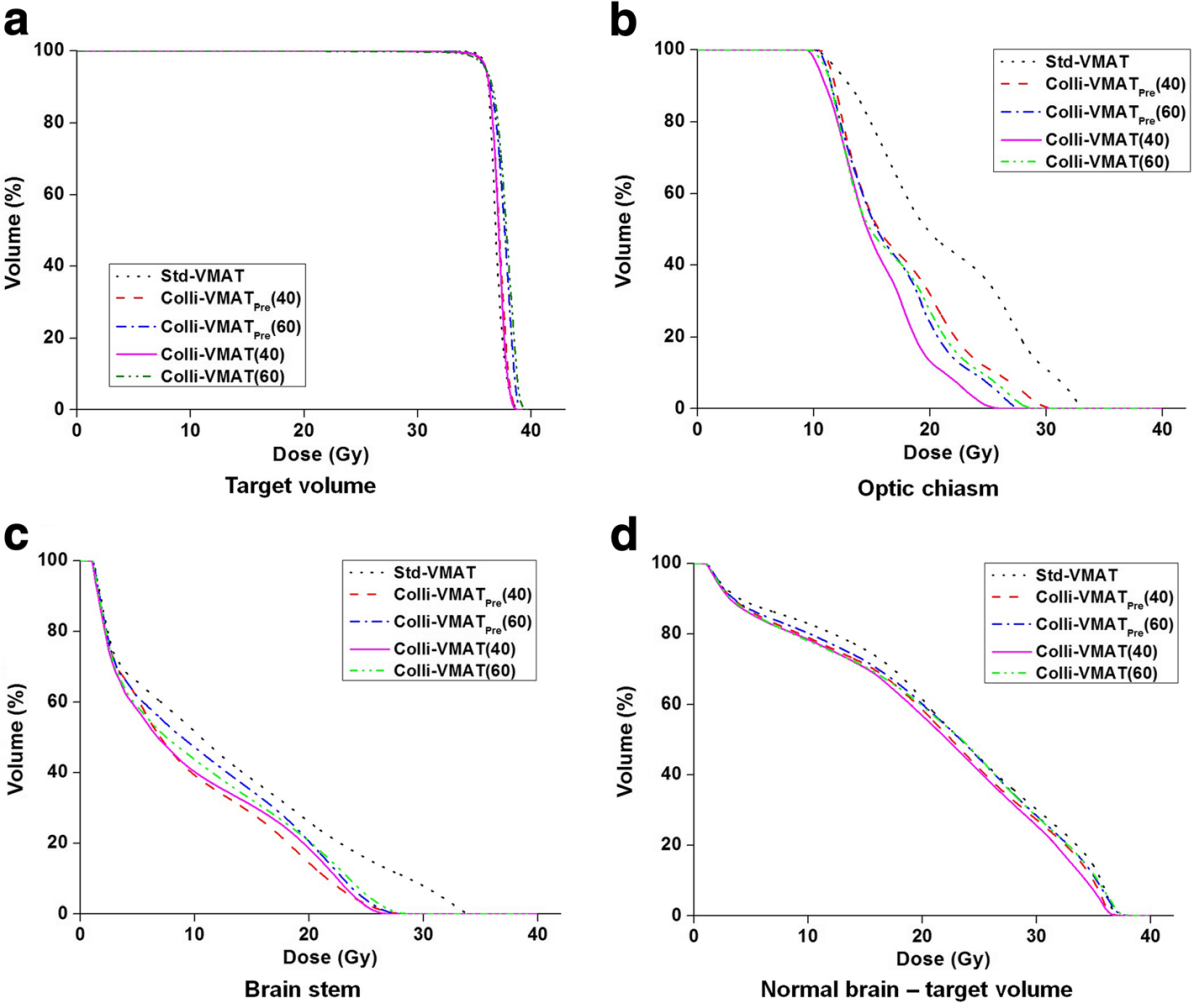
Examples of dose-volume histograms (DVH) of target volumes (**a**), optic chiasm (**b**), brain stem (**c**), and normal brain minus target volume (**d**) for multiple brain targets. The solid lines represent the DVHs of Colli-VMAT plans with an angular section of 40° (Colli-VMAT (40°)). Those of Colli-VMAT plans with angular sections of 60° (Colli-VMAT (60°)), Colli-VMAT plans with angular sections of 40° and 60° in the previous study (Colli-VMAT_Pre_ (40°) and Colli-VMAT_Pre_ (60°)), and Std-VMAT plans are plotted with dash-dot-dotted, dashed, dash-dotted, and dotted lines, respectively

For comparison of Std-VMAT with Colli-VMAT_Pre_ (40°) and Colli-VMAT (40°), most of the dose-volumetric parameters of normal tissues for Colli-VMAT (40°) and Colli-VMAT_Pre_ (40°) were lower than those for Std-VMAT and showed statistical significance. The patient-averaged mean doses for the left parotid gland were similar regardless of the VMAT plans. When comparing Colli-VMAT (40°) to Colli-VMAT_Pre_ (40°), the patient-averaged maximum dose for spinal cord, brain stem, optic chiasm, right optic nerves, and right lens, and the patient-averaged mean doses, V_5 Gy_. V_12 Gy_ and V_15 Gy_ for normal brain without target volumes had slight decreasing tendencies with statistical significance. Figure 3 demonstrates the dose-volume histograms (DVHs) for the representative patient case (Patient #11). There was a slight improvement of the target volume coverage with a reduction of maximum dose in the target volume. For the DVHs of normal tissues, the dose received by all the normal tissues shows a decreasing tendency when Colli-VMAT (40°) was applied. The maximum dose deviation between Colli-VMAT (40°) and Std-VMAT was 7.2 Gy for the DVH of optic chiasm.

The averaged total MUs with respect to Std-VMAT, Colli-VMAT_Pre_ (40°), Colli-VMAT_Pre_ (60°), Colli-VMAT (40°), and Colli-VMAT (60°) were 472 ± 235 MU, 400 ± 154 MU, 419 ± 168 MU, 390 ± 148 MU, and 417 ± 167 MU, respectively, and the *p*-values for for one-way analysis of variance (ANOVA) with Bonferroni multiple comparison of the patient-averaged values of the dose-volumetric parameters were calculated for statistical analysis, as detailed in Table 5. The results of the averaged total MUs were statistically significant except for comparisons between Colli-VMAT_Pre_ (40°) and Colli-VMAT (60°) (*p* = 0.057), and between Colli-VMAT_Pre_ (60°) and Colli-VMAT (60°) (*p* = 0.356). The values of the averaged total MUs for Colli-VMAT_Pre_ plans and Colli-VMAT plans were smaller than those for the Std-VMAT plan, with statistical significance. When the Colli-VMAT (40°) plan was used, the averaged total MU was lowest.

**Table 5:** Patient-averaged values of total monitor units (MUs) and MU reduction with respect to the optimal collimator angles, and *p* values for one way analysis of variance (ANOVA) with Bonferroni multiple comparison of the patient-averaged values of MU

	Std-VMAT	Colli-VMAT_Pre_ (40°)	Colli-VMAT_Pre_ (60°)	Colli-VMAT (40°)	Colli-VMAT (60°)
Average	472 ± 235	400 ± 154	419 ± 168	390 ± 148	417 ± 167
MU reduction (%)	0.0	−15.4	−11.3	−17.4	−11.8
Bonferroni adjusted *p*-values					
Colli-VMAT_Pre_ (40°)	0.004	–	–	–	–
Colli-VMAT_Pre_ (60°)	0.011	0.029	–	–	–
Colli-VMAT (40°)	0.010	0.012	0.046	–	
Colli-VMAT (60°)	0.023	0.057	0.356	0.047	–

## Discussion

**Fig. 4:**
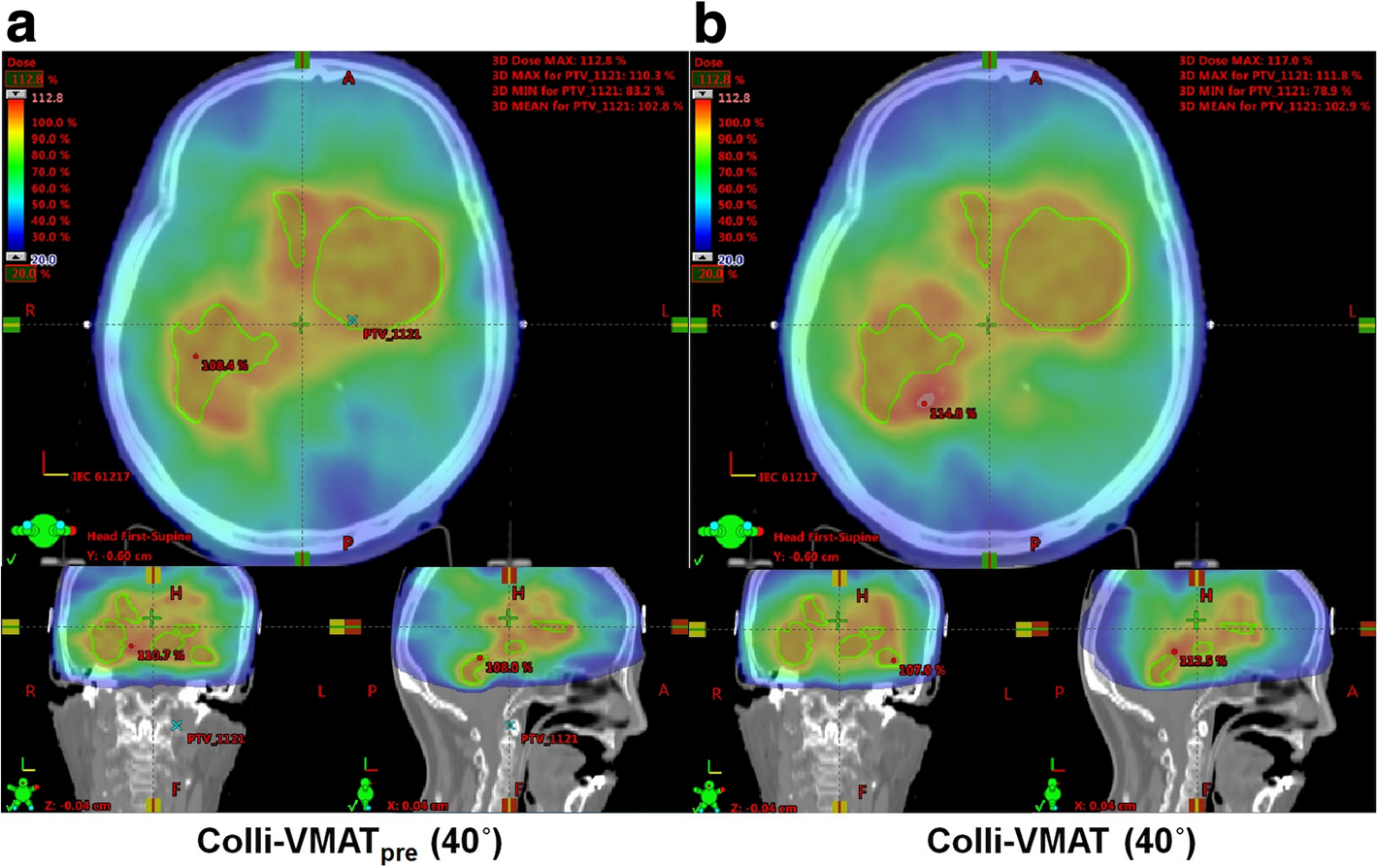
For a representative patient case (patent 6), calculated dose distribution in the axial, coronal, and sagittal views of the volumetric modulated arc therapy (VMAT) plans with the optimal collimator angles with angular sections of 40° in the previous study (Colli-VMAT_Pre_ (40°)) (**a**) and this study (Colli-VMAT (40°)) (**b**). The target volume of Colli-VMAT_Pre_ (40°) and Colli-VMAT (40°), which was the planning target volume (PTV), is shown in a green color

In a previous study, we demonstrated the potential of optimal collimator angles during the VMAT delivery for abdomen, head and neck, and chest cases, all of which had an irregularly shaped target, showing high reduction of the total MUs and improvement of dosimetric plan quality, compared to a fixed collimator angle. In that study, Colli-VMAT_Pre_ plans with angular sections of 40° could cover the large and irregularly shaped target, and by using a DICOM-RT format file of the conformal arc plan with a collimator setting of 0°, the optimal collimator angles could be calculated effectively [16]. However, as mentioned above, MLC positions in the conformal arc plans generated with a collimator setting of 0° could not fully express the target outline. In the case of multiple targets, in particular for brain cases, shapes of the multiple brain targets defined by the MLC positions could be greatly changed according to the collimator settings. In this study, we acquired conformal arc plans with four collimator settings of 0°, 45°, 90°, and 135° to find a realistic outline of the multiple brain targets irrespective of the collimator angles. With this technique, the outlines of multiple brain targets could be properly acquired. DICOM-RT structure files contained point clouds in xyz format to represent the real geometry of target volumes. However, there was a limitation for obtaining the 2D outline of the target volume on any plane defined by BEV. In order to perform 2D interpolation to a 3D surface, several models to generate 3D triangulated meshes should be used, which is complicated and time-consuming. For that reason, conformal arc plans to simply obtain the outline of the target volume were used in this study. For dosimetric evaluation, dose distributions in Colli-VMAT_Pre_ (40°) and Colli-VMAT (40°) for multiple brain targets are shown in Fig. 4. The MLC apertures used in this study to represent realistic brain targets showed dose reduction in normal brain, compared to those in the previous study [16]. The technique proposed in this study could reduce unnecessary dose exposure to the normal brain with similar plan quality. However, the four collimator settings and MLC positions still had limitations that could not fully represent the various shapes and the number of targets. Furthermore, target contouring information will be used when defining the accurate outline of random targets regardless of the shape and the number of targets in our future studies.

Similar to our previous study, the values of field sizes defined by the optimal collimator angles for the Colli-VMAT (40°) plan was decreased compared to those for the Colli-VMAT_Pre_ (40°) and Std-VMAT plans and it was demonstrated that the area size difference between the integrated MLC aperture and collimator settings was minimum using four conformal arc plans. The minimum area size difference and reduction of the total MUs mean that the MLC area exposed by the photon beam was smaller. It was demonstrated that the portion of scatter and leakage radiation to patients in treatment could be reduced due to these effects. Several studies have reported that the secondary cancer risk to patients may be increased by scatter and leakage doses after introducing IMRT and VMAT techniques that use high MUs [20–28]. Therefore, it was critical to reduce the doses from scatter and leakage radiation. By minimizing the area size differences and the total MUs for our study, the clinical advantages of reduction of secondary cancer risk from radiotherapy and comparable dosimetric quality of the VMAT could be expected.

As optimal collimator angles were calculated by using the integrated MLC apertures from the conformal arc plans, the optimal collimator angles could not be reflected properly to that for VMAT plans. Although we demonstrated the dosimetric improvement and MU reduction of VMAT plans with optimal collimator angles for multiple brain targets as well as for irregularly shaped targets, there are limitations in applying this technique to all cases owing to the reasons mentioned above. In addition to this technique, there should be an attempt to find the optimal collimator angles at various treatment sites.

The results in this study show similarity to those of the previous study. It has been demonstrated that the VMAT plans using collimator trajectory achieved improvement of the target coverage and sparing of normal tissue for paraspinal stereotactic body radiation therapy (SBRT), as compared to those with a fixed collimator angle [15]. Several studies have reported that there was improvement on the effect of dynamic collimator angle on the dosimetric quality to correct roll and pitch errors in the head and neck cases, and prostate cases. They have demonstrated that this technique could improve planning target volume (PTV) coverage and decrease the maximum dose to normal organs [10, 14]. Furthermore, dynamic adjustments of collimator angle during the VMAT is the ideal way to improve delivery efficiency and dosimetric plan quality of the VMAT plans. Conformal arc plans generated with four collimator settings suggested in this study were adopted to calculate the optimal collimator angles in the VMAT plans, and the results of this study showed a noticeable improvement for the dosimetric plan quality and reduction of the total MUs as compared to Colli-VMAT_Pre_ plans. If the minimum angular section could be decreased in Eclipse™, the effect of optimal collimator angle suggested in this study on dosimetric quality could be improved. This technique should account for target outline and optimization freedom to shape a desired dose distribution throughout all CPs control, and could be applied in the process of optimization; thus, improved VMAT plans that have better dosimetric plan quality will be generated. This will be investigated in future studies.

## Conclusions

In this study, four collimator settings of the conformal arc plans were utilized to cover the multiple brain targets properly and then Colli-VMAT plans with optimal collimator angles were generated. The results of this study which were dose-volumetric parameters for target volume and normal tissues, field sizes and total MUs were compared with Colli-VMAT plans, Colli-VMAT_Pre_ plans and the Std-VMAT plan. The Colli-VMAT plans with angular sections of 40° have beneficial effects to reduce the total MUs and spare the normal tissues. By using this technique, the Colli-VMAT plans with angular sections of 40° could be clinically suitable for multiple brain targets.

## Abbreviations

AAA: Anisotropic analytic algorithm
ANOVA: One-way analysis of variance
BEV: Beam’s-eye view
CI: Conformity index
Colli-VMAT: VMAT plans with the optimal collimator angles
CP: Control point
D_n%_: Dose received by n% volume of a structure
DVH: Dose-volume histogram
GM: Gradient measure
HI: Homogeneity index
IMRT: Intensity-modulated radiation therapy
MLC: Multi-leaf collimator
MU: Monitor unit
PRO3: Progressive resolution optimizer 3
R_*x*_: Sphere radius of which the volume is the same as the volume of isodose of *x*
Std-VMAT: Conventional VMAT plans with a fixed collimator angle
VMAT: Volumetric modulated arc therapy
V_n Gy_: Absolute volume of a normal brain without target volumes irradiated by at least n Gy
